# Eggshell Waste Valorization: Nutritional Composition, Functional Properties, and Industrial Applications

**DOI:** 10.1002/fsn3.72546

**Published:** 2026-11-03

**Authors:** Farhang Hameed Awlqadr, Iffat Ullah, Muhammad Tayyab Arshad, Mamoona Shehzadi, Ali Ikram, Meerab Sajid, Mohammed Ali Al‐Duais, Md. Sakhawot Hossain

**Affiliations:** ^1^ Food Science and Quality Control, Halabja Technical College Sulaimani Polytechnic University Sulaymaniyah Iraq; ^2^ Department of Pharmaceutical Chemistry, Faculty of Pharmaceutical Sciences Prince of Songkla University Songkhla Thailand; ^3^ Drug Delivery System Excellence Center, Faculty of Pharmaceutical Sciences Prince of Songkla University Songkhla Thailand; ^4^ Functional Food and Nutrition Program, Faculty of Agro‐Industry Prince of Songkla University Songkhla Thailand; ^5^ University Institute of Food Science and Technology The University of Lahore Lahore Pakistan; ^6^ College of Pharmaceutical Science Zhejiang University of Technology Hangzhou People's Republic of China; ^7^ University Institute of Dietetics and Nutrition Sciences The University of Chenab Gujrat Pakistan; ^8^ Department of Biochemistry, Faculty of Science University of Tabuk Tabuk Saudi Arabia; ^9^ Department of Nutrition and Food Technology Jashore University of Science and Technology Jashore Bangladesh; ^10^ Department of Nutrition and Food Engineering Daffodil International University Dhaka Bangladesh

**Keywords:** calcium carbonate, circular economy, eggshell waste, valorization

## Abstract

Eggshell waste, a byproduct of global egg production, is a calcium‐rich secondary resource whose disposal creates environmental and resource recovery challenges. This review critically integrates current knowledge on the composition of the mineral shell and eggshell membrane, pretreatment and conversion routes, resulting material properties, and applications in food, biomedical, environmental, agricultural, and industrial systems. Eggshells predominantly consist of calcium carbonate (CaCO_3_), whereas the membrane contains collagenous proteins, glycosaminoglycan, and other matrix proteins. Cleaning, heat treatment, grinding, calcination, sol–gel conversion, mechanochemical synthesis, microwave‐assisted processing, and microbial bioprocessing can produce eggshell powder, CaO, calcium phosphate bioceramics, adsorbents, catalysts, and composite fillers with application‐dependent properties. Evidence supports calcium fortification with appropriately processed eggshells, experimental use of eggshell‐derived hydroxyapatite and β‐tricalcium phosphate in scaffolds or drug delivery systems, and applications in adsorption, catalysis, mineral fillers, and soil amendment. However, most routes remain at the laboratory or pilot scale, and the environmental superiority of green processes is rarely established by life cycle assessment. Feedstock variability, sanitation, process standardization, energy and chemical demand, regulatory classification, clinical validation, and consumer acceptance remain major barriers. By linking composition, processing, material performance, evidence maturity, and translation requirements, this review identifies priorities for safe and scalable eggshell waste valorization.

## Graphical abstract

This review aims to summarize the composition and functional roles of the shell and membrane, compare conventional, thermal, chemical, mechanochemical, microwave‐assisted, and biological processing routes, link processing conditions to material properties and application performance, distinguish laboratory proof‐of‐concept from pilot, semi‐industrial, or commercial evidence, and identify safety, standardization, regulatory, economic, and sustainability priorities.

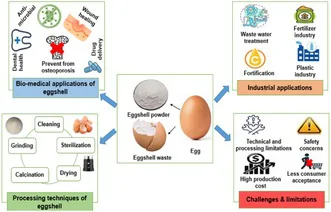

**Abbreviations**
ACC: amorphous calcium carbonate
CaP: calcium phosphate
ESM: eggshell membrane
ESP: eggshell powder
GAGs: glycosaminoglycans
HAp: hydroxyapatite
LCA: life‐cycle assessment
PCL: polycaprolactone
PEF: pulsed electric field
SEM: scanning electron microscopy
TEA: techno economic analysis
TEM: transmission electron microscopy
TGA: thermogravimetric analysis
XRD: X‐ray diffraction
β‐TCP: beta‐tricalcium phosphate

## 1 Introduction

Global egg production generates substantial quantities of eggshell residues from households, food service operations, hatcheries, and egg processing facilities (Zeng et al. 2026). Generally, this waste is dumped in landfills without treatment, resulting in sustainability issues such as soil and water contamination, organic decomposition, and greenhouse gas emissions, and underutilization of a calcium‐rich biomass. As they are mainly made of calcium carbonate, their improper disposal causes economic and ecological concerns due to their role in waste build‐up and the inability to use potentially useful resources (Abdelgalil et al. 2024; Babalola and Wilson 2024; Ferraz et al. 2018; Ng and Wong 2024). Growing environmental concerns associated with eggshell waste disposal have driven research into valorization strategies that convert this biomass into valuable products within a circular economy. Valorization is a process where the waste eggshell is treated and recycled so that the useful components can be obtained or it can be reused as a useful material with less impact on the environment. Indicatively, high‐temperature calcination of eggshells generates calcium oxide (lime), which is a major industrial chemical. Research has demonstrated that lime made using eggshells is equivalent in terms of reactivity to commercial limestone with the possibility of partial replacement in the manufacture of industrial lime, which has economic and environmental advantages of reducing the use of mined limestone and redirecting the waste of eggshells in landfills (Ferraz et al. 2018).

Processing intensity should be matched to the intended product. Washed, sanitized, dried, and milled eggshell powder (ESP) may be used as a calcium‐rich ingredient or filler when purity and particle size are controlled. High‐temperature calcination converts CaCO_3_ to calcium oxide (CaO) according to CaCO_3_(s) → CaO(s) + CO_2_(g), producing a reactive material for lime or catalysis but requiring substantial heat and releasing process CO_2_. Chemical, mechanochemical, and hydrothermal or microwave‐assisted routes can convert eggshell‐derived calcium into nano‐CaO or calcium phosphates. Their selected modular synthesis allows adjusting the properties of adsorption, which enhances their adaptability and facilitates cleaner production strategies by recycling the waste eggshells into high‐value useful materials (Babalola and Wilson 2024; Ferraz et al. 2018).

Biological processing offers a distinct route but must be described precisely. Abdelgalil et al. (2024) reported acid‐phosphatase production by *Bacillus sonorensis* in a fermentation system containing ESP‐derived minerals. The microorganism produced the enzyme using an eggshell‐containing medium; the shell was not directly converted into an enzyme. Such recovery can support circular resource strategies, but circularity alone does not establish environmental benefits without data on yield, energy, water, reagents, emissions, and residual streams (Abdelgalil et al. 2024). These diversified valorization technologies are examples of integration into the principles of a circular economy, whose priorities lie in the reduction of waste, recovery of resources, and sustainable material cycles (Mngadi et al. 2025; Ng and Wong 2024).

In addition to the environmental advantages, valorization of eggshell waste by thermal, chemical, or biochemical treatment can improve the sustainability of an industry, not only by helping industrial processes not to pollute the environment, but also by reducing dependence on non‐renewable resources and generating economic benefits, such as reduced cost of lime production or new pollution control material development (Digitemie et al. 2025; Tawo and Mbamalu 2025).

Existing reviews provide valuable, application‐specific perspectives. Quina et al. emphasized industrial uses and legal considerations; Baláž et al. (2019) focused on materials science, catalysis, pharmaceutical applications, and mechanochemistry; Babalola and Wilson reviewed eggshell‐based bio composite adsorbents; and Torres Gouveia et al. concentrated on medical biomaterials. A remaining need is to connect the distinct compositions of the mineral shell and organic membrane with route‐specific processing changes, measurable material properties, application evidence, and the practical requirements for translation. This gap is important because claims derived from one processed material, for example, CaO nanoparticles or antibiotic‐loaded hydroxyapatite (Hap), cannot automatically be attributed to raw eggshell (Babalola and Wilson 2024; Baláž et al. 2021; Quina et al. 2017; Torres Gouveia et al. 2025).

This review aims to (i) summarize the composition and functional roles of the shell and membrane; (ii) compare conventional, thermal, chemical, mechanochemical, microwave‐assisted, and biological processing routes; (iii) link processing conditions to material properties and application performance; (iv) distinguish laboratory proof‐of‐concept from pilot, semi‐industrial, or commercial evidence; and (v) identify safety, standardization, regulatory, economic, and sustainability priorities. Its contribution is to evaluate the full continuum of composition, processing, properties, applications, and translation across nutritional, biomedical, environmental, agricultural, and industrial uses.

## 2 Eggshell Membrane: Composition and Properties

### 2.1 Structural Characteristics

The eggshell is a hierarchical mineral organic composite comprising an external cuticle, calcified shell layers dominated by the palisade region, a mammillary interface, and inner and outer shell membranes. Each layer has a specific structural and functional role.

#### 2.1.1 Cuticle

The cuticle is the outermost tissue, a non‐calcified organic film mostly composed of glycoproteins, lipids, and small sphere‐shaped nanostructures. It creates a protective barrier that minimizes bacterial adhesion and trans‐shell entry, while also regulating the conductance of water vapor and ultraviolet light reflection. The morphology of the cuticle is known to vary greatly across the species of birds, and this is an indication of adaptation to various nesting conditions. Experimental elimination of the cuticle leads to high bacterial colonization and high UV reflectance, highlighting its protective role (D'Alba et al. 2017).

#### 2.1.2 Palisade Layer

Under the cuticle is the palisade layer, which is the major calcified part of the shell. It consists of columnar, closely packed calcite crystals in a vertical orientation, which makes a hard and tough matrix. Certain soluble matrix proteins inside the palisade layer have molecular weights of approximately 54, 33, 22, and 14 kDa, which are purportedly penetrating the calcite crystal nucleation and development, hence affecting the shell design and mechanical stability. The crystal size and orientation in this layer vary, and this variation has a direct impact on shell quality and fracture resistance (Hincke et al. 1992).

#### 2.1.3 Mammillary Layer

The layer that lies under the palisade layer is the mammillary layer, which is composed of cone‐shaped calcite structures commonly known as mammillary knobs. These knobs act as sites of crystal growth and hold the mineral shell attached to the underlying organic membranes. It has specialized matrix proteins and the prominent 81 kDa calcium binding protein and 38 and 54 kDa proteins that are concentrated in this layer direct mineralization. A high density and consistency of mammillary knobs are important for shell strength because the lower mammillary density of the shell reduces shell membrane interface strength and decreases eggshell mechanical integrity, which is evident in aged hens (Benavides‐Reyes et al. 2021; Hincke et al. 1992; Park and Sohn 2018).

#### 2.1.4 Eggshell Membrane

The eggshell membrane (ESM) is the innermost layer and a fibrous organic structure mainly composed of collagen‐like proteins and glycosaminoglycans (GAGs) organized into multilayered membranes. It is the substrate on which mineral deposition occurs and selective transport of the gases and ions required to support embryonic development occurs in birds. The same types of layered fibrous membranes are seen in reptilian eggs, indicating that this architectural component is evolutionarily preserved (Packard and Demarco 1991; Packard and Packard 1979). A combination of these layers forms a complex biomineralized composite that offers a balance in mechanical strength, defense against microbes and environmental stressors, and physiological processes that are critical to embryo viability. The interaction between the organic matrix proteins, mineral crystal morphology, and layer microstructure is the key to eggshell development and its functionality. Alterations in the structure of any given layer, including the cuticle thickness or the architecture of the mammillary layer, can have a significant influence on the overall quality and strength of the eggshell (Benavides‐Reyes et al. 2021; D'Alba et al. 2017; Hincke et al. 1992).

### 2.2 Bioactive Components

Eggshells contain bioactive molecules, including collagen glycosaminoglycan (GAGs), and a wide range of matrix proteins, all of which are essential for the formation, mineralization, mechanical strength, and antimicrobial protection of eggshells.

#### 2.2.1 Collagen

Collagenous proteins provide structural organization within the native shell membranes. Type I and Type X collagen have been identified in avian ESM, although their biological function in eggshell formation should be distinguished from the experimental use of processed ESM as a biomaterial. Shell matrix proteins, such as ovocleidins and ovocalyxins, contribute to mineral‐organic interactions. These constituents influence nucleation, crystal morphology, interfacial organization, and mechanical behavior during biomineralization (Carrino et al. 1996; Sharifi et al. 2022).

#### 2.2.2 Glycosaminoglycans (GAGs)

These are linear polysaccharides in the organic matrix of the eggshell, which have discrete localization patterns and play a role in the structure and mineralization of the eggshell. In particular, keratin sulfate and dermatan sulfate are the most noticeable, the latter being highly concentrated in the palisade layer, which is the major calcified area, whereas the former is commonly linked with the mammillary bodies. These sulfated GAGs interact with calcium ions and regulate the morphology of calcium carbonate crystals, resulting in smaller and more rounded crystals that resemble those of native eggshell minerals. Moreover, the anionic character of dermatan sulfate proteoglycans indicates that they may be involved in regulating crystal nucleation, similar to other biomineralized tissues. The variation in GAGs in species and eggshell layers indicates functional specialization in controlling biomineralization, ion binding, and mechanical properties. In addition to eggshells, GAGs are essential ECM components that have central mechanosensory and regulatory roles in tissues, which is why they have wide biological significance (Berdiaki et al. 2024; Carrino et al. 1996; Panheleux et al. 1999).

#### 2.2.3 Proteins

Matrix proteins in the eggshell make up a complex array of eggshell‐specific proteins, such as ovocleidins, ovocalyxins, and other structural and regulatory proteins. Proteomic and genomic studies have demonstrated that these proteins have two roles: they mediate eggshell mineralization, which is the regulation of calcium carbonate deposition and crystal growth, and they also mediate antimicrobial invasion of the developing embryo. These matrix proteins have species‐specific variations but have common fundamental functions, which are the basis of eggshell strength and biological defense. Osteopontin and lysozyme are important matrix proteins that aid mineralization and exhibit antimicrobial effects, which indicates the multifunctional nature of the eggshell organic matrix (Panheleux et al. 1999; Rose and Hincke 2009; Sun et al. 2013). The physical scaffold of mineral attachment and growth is made of collagen fibers, the morphology of crystals and ionic interactions is regulated by GAGs, and finally the pattern of mineral deposition and antimicrobial defense is coordinated by matrix proteins. These parts are synergistically active to create a robust and functional eggshell that satisfies physiological needs of embryo development and environmental stressors. Therefore, the bioactivity of eggshells molecular components lies at the center of their structure, composition and functionality, which have great consequences in both avian reproductive biology and biomaterials science and regenerative medicine (Carrino et al. 1996; Panheleux et al. 1999; Rose and Hincke 2009; Sharifi et al. 2022).

### 2.3 Functional Properties

The bioactive constituents of eggshells have multifaceted functional attributes that are essential for eggshell formation, mechanical strength, antimicrobial protection, and the general control of the mineralization process. The eggshell itself is a biomineral compound that is mostly made of calcium carbonate (calcite form) embedded in a complex organic framework that holds more than 900 known proteins. These parts coordinate the biomineralization process, which is tightly controlled to create the hierarchical structure of the eggshell with optimized mechanical characteristics (Gautron et al. 2021). The major protein domains, including calcium‐binding, vesicle binding, and protein interaction motifs, are used to mediate interactions with the mineral phase. Recent discoveries emphasize the development of vesicle‐mediated transport of amorphous calcium carbonate (ACC), in which glycoproteins EDIL3 and MFGE8 retrieve calcium‐rich extracellular vesicles and direct their delivery to the mineralization front to maintain a sustained and regulated supply of mineral ions to the shell formation (Le Roy et al. 2021; Stapane et al. 2019).

Matrix proteins not only control biomineralization but also contribute to the mechanical strength of eggshells. Their action with calcite crystals affects the crystal size, orientation, and stability, which is the basis of the toughness of the shell and fracture resistance. Proteoglycans and other matrix molecules regulate the hierarchical assembly of mineral and organic phases, which contributes to the structural integrity necessary to support the developing embryo (Gautron et al. 2021). In addition to mineralization and mechanical functions, the eggshell matrix harbors a wide array of antimicrobial proteins and peptides (AMMPPs), including defenses, lectins, ant proteases, and chelators, which offer innate immune defense (inhibition of pathogen invasion). Some antimicrobials have dual functions as shell microstructure and texture modulators, and during embryonic development, when the shell mineral is sequentially resorbed, these proteins can be released to strengthen egg defense, thereby preserving antimicrobial protection despite weakening physical barriers (Moreau et al. 2022). Studies on gene expression in eggshell calcification have determined groups of proteins that are actively overexpressed during the eggshell calcification process, including proteins implicated in the movement of mineral ions, molecular chaperoning, biomineralization, antimicrobials, and protease functions. Other proteins, including EDIL3, are highly controlled in space and time and serve to provide molecular cues during vesicle transport and mineral deposition. Their clear expression and activity play a basic role in triggering, regulating, and healing shell mineralization to maintain high quality in eggshells (Brionne et al. 2014; Le Roy et al. 2021; Yan et al. 2017).

Together, the mineral shell and organic membrane form a multifunctional composite in which structure, mineralization, mechanics, and defense are coupled. Therefore, valorization should begin by deciding whether the target is the mineral fraction, the membrane fraction, or a deliberately combined material, because processing conditions that activate CaCO_3_ may destroy membrane bioactivity (Brionne et al. 2014; Gautron et al. 2021; Le Roy et al. 2021; Moreau et al. 2022).

## 3 Processing and Valorization Techniques of Eggshell

### 3.1 Pre‐Treatment Methods

Pretreatment comprises collection, removal of residual egg contents, washing, application‐specific sanitation, drying, membrane separation where required, and size reduction. The sanitation step must reduce microbial risk; if membrane proteins are the target, unnecessary heat or chemical severity should also be avoided. Dry grinding or ball milling reduces particle size and can modify the microstructure; however, it does not convert CaCO_3_ into CaO or HAp (Bartter et al. 2018; Ononiwu and Akinlabi 2020).

#### 3.1.1 Cleaning

Eggshells are to be cleansed to eliminate the remaining organic material, which could include membrane fragments, proteins, and possible microorganism contamination. Proper cleaning will guarantee a cleaner source of calcium carbonate and improve downstream processing. Although there are some mechanical or chemical methods of cleaning, the general methods include washing with water and detergents, followed by manual or mechanical extraction of the membranes. This is the primary step before sterilization and grinding of materials into powders or composites to enhance the material consistency and adsorption capacity in the field of environment or agriculture (Babalola and Wilson 2024).

#### 3.1.2 Sterilization Process

Sterilization processes are essential for eliminating microbial hazards and extending the shelf life of eggshell waste. Although conventional thermal treatments, such as dry or moist heat, are commonly applied, they can degrade the organic matrix and alter the mineral structure unless processing conditions are strictly controlled, which may compromise mechanical strength or adsorption capacity. Recent developments in sterilization aim to achieve rapid microbial inactivation while minimizing damage to the eggshell structure and its bioactive components. These include chemical agents with shorter exposure times (e.g., ortho‐phthalaldehyde), antimicrobial coatings, and plasma‐based systems. Additionally, novel non‐thermal technologies, such as pulsed electric field (PEF) treatment electroporate cell membranes without high heat, preserving both the structural integrity and bioactive constituents of the eggshell (Capodaglio 2021; Rutala and Weber 2001).

#### 3.1.3 Drying Methods

Drying methods are used to stabilize the cleaned and sterilized eggshell waste by lowering the moisture level to prevent microbial growth and ease storage and handling over a long period. Drying can be done using several different methods, including conventional oven drying, freeze‐drying (lyophilization), solar drying, air drying, and new bio‐drying methods. Moderate temperatures in oven drying are effective in removing moisture while retaining mineral contents but can destroy thermolabile organic molecules when the heating duration is too long or the heating temperature is too high. Freeze drying is the most effective method of preserving organic bioactive fractions and microstructure but is more expensive and energy intensive. Other inexpensive methods, such as solar and air drying, rely on climatic conditions. Bio‐drying applies regulated microbial activity under aerobic conditions to dry the moisture with minimum environmental impact, which is in line with the objectives of the circular economy. The drying process determines the physical properties of the drying powders: morphology of the particles, surface area, and porosity, which also determines the adsorption capacity and functional performance of ESP in environmental remediation or agricultural applications (Chaji et al. 2024; Tan et al. 2022).

In short, the full pre‐treatment of eggshell waste is split into three steps: cleaning of their surfaces to eliminate organic residues and contaminants, proper sterilization to ensure that it is microbiologically safe and that materials remain stable, and finally, proper drying to keep the materials intact and to the maximum possible valorization. These procedures need to be streamlined to harmonize microbial control, conservation of the mineral organic matrix, and cost‐effectiveness depending on the desired downstream use. Improvements in the sterilization of eggshells and the use of specialized methods to dry them (particularly non‐thermal technologies) have shown the potential to expand the valorization of eggshell waste in the context of more sustainable circular economies (Babalola and Wilson 2024; Capodaglio 2021; Rutala and Weber 2001; Tan et al. 2022).

### 3.2 Conversion Processes

Eggshell conversion processes are mainly carried out to convert calcium carbonate (CaCO_3_) in eggshells into calcium oxide (CaO) and powders with specific particle sizes suitable for diverse applications.

#### 3.2.1 Calcination

Calcination converts the dominant CaCO_3_ phase to CaO: CaCO_3_(s) → CaO(s) + CO_2_(g). Ferraz et al. calcined eggshells at 1000°C and obtained a highly reactive lime suitable for the application studied. Other studies used lower or different time–temperature profiles; therefore, phase conversion should be verified by X‐ray diffraction (XRD) and, where appropriate, thermogravimetric analysis (TGA). The advantages include a simple transformation and high reactivity of CaO; the disadvantages include the heat demand, process CO_2_, possible sintering/agglomeration, and loss of organic membrane constituents (Ferraz et al. 2018).

#### 3.2.2 Grinding and Powder Formation

After the stages of calcination, grinding and powder forming are used to make fine powders which can be used in the industry, for example, catalysts, composite fillers, or energy storage materials. The dispersion and size of particles can be controlled using mechanical grinding techniques, including ball milling and manual grinding. For example, mechanical grinding and compression molding have been used to manufacture eco‐composites made of ESP and polyethylene glycol. The powders obtained retain the organic or inorganic structure, which can be supportive of thermal stability and latent heat storage (Ben Aribia et al. 2024). In summary, eggshell valorization through calcination can be used to obtain high‐purity CaO by subjecting CaCO_3_ to high temperatures for sufficient periods. Following grinding defines powder properties required to perform various functions in their use. The effectiveness of these conversion parameters is properly optimized to guarantee the purity of the material phases, control of particle sizes, and physical properties that are specific to their environmental, catalytic, or composite applications (Fayyazi et al. 2018; Habte et al. 2019; Sahadat Hossain et al. 2023).

### 3.3 Advanced Technologies

Advanced processing routes can convert eggshell waste into nano‐calcium materials; high‐temperature calcination is a thermal conversion method, whereas genuinely greener approaches aim to reduce energy demand, avoid hazardous reagents, or use biological/low‐temperature processing.

#### 3.3.1 Calcination‐Based Production of CaO Nanoparticles

Eggshells primarily consist of calcium carbonate (CaCO_3_). During calcination, CaCO_3_ thermally decomposes into calcium oxide (CaO) and carbon dioxide (CO_2_).

For example, calcination of chicken eggshells at 700°C for 7 h produced spherical, single‐crystal CaO nanoparticles with diameters of approximately 5–30 nm and an average crystallite size of approximately 20 nm. The resulting CaO nanoparticles showed antibacterial activity against *Staphylococcus aureus* , *Klebsiella pneumoniae* , and *Escherichia coli* , antifungal activity against *Candida albicans* , and high removal efficiencies for Pb^2+^, Cd^2+^, Cr^2+^, and Hg^2+^ from aqueous media under optimized adsorption conditions. Although this approach valorizes a biowaste feedstock, it requires prolonged high‐temperature heating and releases CO_2_ during CaCO_3_ decomposition. Therefore, despite its waste‐valorization benefits, the process has substantial energy requirements and should be evaluated critically before being classified as an environmentally sustainable synthesis method.

In contrast, microwave‐assisted, mechanochemical, biological, and low‐temperature wet‐chemical routes may reduce processing time, thermal demand, and/or use of harsh reagents. Their environmental benefits should be demonstrated using energy use data, reagent assessment, and preferably life cycle analysis rather than assumed solely because eggshell waste is used as the starting material (Hemmami et al. 2024).

#### 3.3.2 Sol–Gel Method

The sol–gel method has been investigated for the synthesis of nano calcium oxide from eggshell waste. In the reported procedure, raw eggshell was dissolved in hydrochloric acid to obtain a calcium chloride solution, followed by dropwise addition of sodium hydroxide to form a calcium hydroxide gel. Spherical CaO nanoparticles with diameters of approximately 50–198 nm were subsequently obtained after calcination at 900°C (Habte et al. 2019). The sol–gel route offers advantages such as relatively simple precursor processing and control over particle formation, and the wet‐chemical steps can be conducted under comparatively mild temperature and pressure conditions. However, the final calcination at 900°C represents a substantial thermal energy requirement and should be included when assessing the environmental performance of the complete synthesis.

#### 3.3.3 Microwave Irradiation

Microwave‐assisted chemical synthesis was used to prepare nano‐hydroxyapatite (HAp) from decarbonized eggshells. First, eggshell calcium carbonate (CaCO_3_) reacted with sodium hydroxide (NaOH) under alkaline conditions to form calcium hydroxide [Ca(OH)_2_], while the carbonate fraction was retained as sodium carbonate (Na_2_CO_3_):CaCO_3_ + 2NaOH → Ca(OH)_2_ + Na_2_CO_3_. The resulting Ca(OH)_2_ was then mixed with ammonium dihydrogen phosphate (NH_4_H_2_PO_4_), and the reaction mixture was adjusted to approximately pH 11 to supply calcium and phosphate ions for HAp formation. Subsequently, the mixture was subjected to microwave irradiation at 2.45 GHz and 800 W for 5 min. This combined chemical, microwave route produced rod‐like nano‐HAp crystals with an approximate crystallite size of 28 nm. Therefore, microwave irradiation should be described as assisting the final formation and crystallization of HAp, rather than as a standalone conversion process. The retention of carbonate as Na_2_CO_3_ may avoid direct CO_2_ release associated with conventional calcination; however, comparative life cycle assessment is needed to confirm an overall environmental advantage (Mahmoud 2024).

Green synthesis strategies are emerging beyond eggshells and towards the use of plant‐mediated fabrication of nanoparticles, in which biological extracts are used as reducing, stabilizing, and capping agents for metal and metal oxide nanoparticles. They are environmentally friendly, less dangerous to human health, and reduce the toxicological problems of the traditional synthesis of chemicals. Although these methods using plant extracts have been widely used to produce a wide range of nanoparticles, such as silver and spinel ferrite, their theology can similarly be used to produce nanotechnology‐based materials, such as nano‐calcium, in an eco‐friendly manner, in addition to many other nanoparticles, which would enable the creation of materials with a reduced environmental impact (Rathod et al. 2024; Wani and Suresh 2022).

These emerging technologies provide different opportunities for valorizing eggshell waste into nano calcium materials with controlled particle size and morphology. However, their environmental performance should not be assumed solely from the use of a waste feedstock or from the designation of a process as “green.” Calcination‐based and sol–gel routes may involve substantial thermal and chemical inputs, respectively, whereas microwave, mechanochemical, and biological approaches may reduce particular energy or reagent requirements under specific conditions. Consequently, the sustainability of each route should be evaluated using the complete process boundary, including pretreatment, chemical consumption, energy use, emissions, waste generation, and downstream processing. Comparative life cycle and techno economic assessments are therefore needed to determine whether a given route provides a genuine environmental or economic advantage at scale (Habte et al. 2019; Hemmami et al. 2024; Mahmoud 2024; Wani and Suresh 2022).

No single processing route is optimal for all applications. Route selection should be based on the target phase and function, required purity and particle size, retention or removal of membrane constituents, microbial controls, yield, energy and reagent use, water and effluent generation, equipment requirements, and achievable scale. Environmental or green superiority should be supported by a defined life‐cycle comparison, and commercial feasibility should be supported by techno‐economic analysis (TEA). Table 1 outlines the comparison of the principal processing routes used for eggshell waste valorization.

**TABLE 1:** Comparison of principal processing routes used for eggshell waste valorization.

Route	Representative process and product	Advantages	Limitations	References
Direct powder processing	Washing, sanitation, drying and grinding produce CaCO_3_ rich eggshell powder for food fortification, fillers, adsorbents and agricultural applications.	Simple process; limited chemical use; retains the native CaCO_3_ phase; comparatively low processing temperature.	Residual membrane, microorganisms and particle size variability must be controlled; raw powder may not meet biomedical or high‐purity requirements.	Quina et al. (2017), Vandeginste (2021)
Calcination	Heating at approximately 600°C–1000°C converts CaCO_3_ into CaO. Gergely et al. used 900°C for 3 h before subsequent calcium‐phosphate processing.	Produces reactive CaO; removes most organic matter; suitable for catalysts, adsorbents and calcium phosphate synthesis.	High energy consumption; direct CO_2_ generation; particle sintering or agglomeration; careful temperature control is required.	Baláž et al. (2021), Gergely et al. (2010)
Sol–gel synthesis	Eggshell‐derived calcium precursor is mixed with a phosphate precursor under controlled pH, followed by gel formation, aging, drying and heat treatment. Azis et al. produced HAp particles of approximately 35–54 nm.	Molecular‐level mixing; good chemical homogeneity; control of Ca/P ratio, composition and particle characteristics.	Multistep process; requires acids, bases and phosphate reagents; extended aging and drying; final heat treatment may still be necessary.	Azis et al. (2018)
Microwave‐assisted synthesis	Eggshell‐derived Ca(OH)_2_ is combined with NH_4_H_2_PO_4_ at approximately pH 11 and irradiated at 2.45 GHz and 800 W for 5 min, producing rod‐like HAp crystals of approximately 28 nm.	Very short reaction time; rapid and relatively uniform heating; production of nanoscale HAp.	Requires chemical pretreatment and specialized equipment; microwave uniformity and reactor penetration can become challenging during scale up; environmental advantages require life cycle confirmation.	Mahmoud (2024)
Mechanochemical synthesis	Calcined eggshell and phosphoric acid are processed by ball or attritor milling. In one study, attritor milling was conducted for 5 h, while ball milling required approximately 10 h.	Intimate solid mixing; reduced solvent demand; particle size reduction and reaction can occur simultaneously.	Milling may require substantial electrical energy; contamination from milling media is possible; localized heating and equipment wear can affect product quality.	Baláž et al. (2021), Gergely et al. (2010)

## 4 Medicinal and Biomedical Applications of Eggshell

Calcium materials derived from eggshells have also garnered increasing interest as multifunctional agents in calcium supplementation, bone regeneration, antimicrobial effects, wound healing, supporting bone tissue engineering applications, and as drug delivery vehicles owing to their natural biocompatibility and chemical resemblance to native bone minerals.

### 4.1 Calcium Supplementation and Bone Regeneration

Eggshells are rich in calcium carbonate (CaCO_3_) and can serve as an inexpensive, biogenic calcium source for the synthesis of calcium‐phosphate biomaterials. Importantly, HApand β‐tricalcium phosphate (β‐TCP) do not occur naturally in eggshells. Instead, they are synthesized by reacting eggshell‐derived calcium with a phosphorus‐containing precursor. Typically, ESP is first thermally treated to convert CaCO_3_ into calcium oxide (CaO). The eggshell‐derived CaO or a calcium‐containing intermediate such as Ca(OH)_2_ is then reacted with phosphate precursors, such as dicalcium phosphate dihydrate (CaHPO_4_·2H_2_O), calcium pyrophosphate (Ca_2_P_2_O₇), phosphoric acid, or ammonium dihydrogen phosphate. Subsequent wet chemical processing, hydrothermal treatment, mechanochemical milling, or solid‐state heat treatment produces HA [Ca_10_(PO_4_)_6_(OH)_2_], β‐TCP [β‐Ca_3_(PO_4_)_2_], or biphasic calcium phosphate containing both phases. The final phase composition depends on the calcium‐to‐phosphorus ratio, precursor type, pH, temperature, and heat treatment conditions.

Because HA and β‐TCP resemble the inorganic mineral component of bone, eggshell‐derived calcium‐phosphate materials have potential for bone defect repair and tissue‐engineering scaffolds. Their suitability depends on confirmed phase purity, porosity, mechanical performance, degradation behavior, cytocompatibility, and osteogenic response. Thus, eggshell valorization offers a sustainable alternative calcium feedstock; however, biomedical claims should be supported by appropriate biological and scaffold‐performance studies (Chen et al. 2023; Sergey 2018; Torres Gouveia et al. 2025).

### 4.2 Antimicrobial Properties

Reported antimicrobial effects are mainly associated with eggshell‐derived HAp nanoparticles (HAp‐NPs), particularly when these particles are used as carriers for antimicrobial drugs or incorporated into antimicrobial composite systems. The nanoscale size, surface characteristics, and drug‐loading capacity of HAp‐NPs can support localized delivery and sustained release of therapeutic agents (Ganesan et al. 2022). For example, vancomycin‐loaded eggshell‐derived HAp nanoparticles showed improved antibacterial performance against *Escherichia coli* and *Staphylococcus aureus* compared with vancomycin alone in the reported in vitro study. This effect should be attributed primarily to the antibiotic and its controlled release from the HAp carrier, rather than to eggshell‐derived HAp alone. Similarly, the antibacterial activity reported for drug‐loaded eggshell‐derived HA systems, such as etoposide‐loaded HA, reflects the combined function of the bioactive drug and the HAp delivery matrix (Ain et al. 2019).

Chitosan calcium phosphate composites may provide an additional antimicrobial strategy because chitosan can possess antimicrobial activity, while HAp contributes bioactivity and osteoconductive properties. However, the antimicrobial performance depends on the composition, drug loading, particle size, microbial strain, assay conditions, and release behavior. Therefore, eggshell‐derived HA‐based materials should be described as promising platforms for localized antimicrobial drug delivery and bone‐related biomedical applications, subject to appropriate in vitro and in vivo validation (Ain et al. 2019; Mawazi et al. 2024; Sultankulov et al. 2019).

### 4.3 Wound Healing and Tissue Regeneration

Eggshell‐derived materials are most relevant to bone and dental tissue engineering after conversion of eggshell calcium carbonate into HAp, β‐tricalcium phosphate (β‐TCP), or related calcium phosphate phases, which resemble the mineral component of natural bone and provide bioactive, osteoconductive surfaces that support cellular attachment, proliferation, and differentiation (Swarup et al. 2025). Eggshell‐derived HA can be incorporated into porous polymeric scaffolds, such as polycaprolactone (PCL), gelatin, collagen, or polyurethane, to combine the bioactivity of calcium phosphate with the flexibility and processability of polymers. In such composites, the scaffold pore architecture and polymer ceramic composition can be tailored to support bone defect repair. Eggshell‐derived HA may also serve as a carrier for locally delivered drugs; however, the drug release and antimicrobial effects depend on the incorporated therapeutic agent and scaffold design (Hajiali et al. 2017; Marszalik et al. 2025; Torres Gouveia et al. 2025). Table 2 shows the natural components associated with eggshells and their role in the body and bone health.

**TABLE 2:** Natural components associated with eggshells and their role in the body and bone health.

Natural components	Significance in the body	Eggshell association	References
Collagen Type I	Plays a crucial part in improving toughness and its ability to absorb energy, whereas the mineral content of the bone mostly affects its stiffness	The thick outer and thin inner layers of hen eggshell membranes have been found to contain collagen‐like proteins that resemble types I and V collagens	Viguet‐Carrin et al. (2006)
Fibronectin and thrombospondin	Matrix glycoproteins that attach to integrins and other extracellular matrix elements, such as fibrin, and collagen	The eggshell membrane, which is mostly composed of around 90% protein and contains complex polysaccharides like glycosaminoglycans and N‐glycans shares structural similarities with the extracellular matrix of bone. It includes ADAMs enzymes with a thrombospondin Type 1 motif, glycoproteins, calcium‐regulating proteins, cysteine‐rich proteins and Type X collagen	Rønning et al. (2024)
Keratin sulphate and chondroitin sulphate	Aid in the adherence and attachment of osteoblastic cells to the extracellular matrix	Glycosaminoglycans and proteoglycans are involved in the mineralization process, according to comparative studies of mineralization models like bone and teeth. The highly sulfated keratin sulphate proteoglycan mammalian which is concentrated in mammillae in the mammillary layer, indicated that keratin sulphate plays a critical function in maintaining structural integrity and promoting mineralization within the eggshell matrix	Nakamura et al. (2007)
Osteopontin	Increase osteoblast adherence to the matrix and makes it easier for bonds with hydroxyapatite to form	The oviduct and eggshell of birds have been found to contain osteopontin	Fernandez et al. (2003), Neunzehn et al. (2015)
Osteonectin	It is thought to act as a bone‐specific nucleator of mineralization due to its strong affinity for both collagen and mineral	Osteonectin also known as SPARC is a 34 kDa glycoprotein that is moderately abundant in mammillary cones Osteonectin which was first discovered in the extracellular matrix of the bones, binds hydroxyapatite calcium crystals and collagen type‐I in vitro, indicating that it plays a part in starting the synthesis of minerals and encouraging crystals attachment to the collagenous matrix. Its presence in eggshell mammillary cones suggests a potential connection to crystal attachment and mineralization in the eggshell structure	Young (2003), Rose‐Martel et al. (2015)
Sialoprotein in bones	Arginine, glycine, aspartic acid are cell binding proteins that appears late in the osteoblast phenotypic development process	The eggshell matrix included the bones sialoprotein	Malaval et al. (2008), Rose and Hincke (2009), Horvat‐Gordon et al. (2008)

### 4.4 Drug Delivery Systems

Eggshell‐based HAp and calcium phosphate nanomaterials are perfect systems to load and release therapeutic agents based on controlled delivery, improve bioavailability, and target drug delivery. The porosity and surface characteristics of these biomaterials promote high drug loading factors and release kinetics. For example, eggshell‐based vancomycin‐loaded HAp nanoparticles have been shown to have better antibiotic release and increased antibacterial properties. Bone regeneration and drug delivery calcium phosphate scaffolds are also often functionalized to undergo both bone regeneration and drug delivery as dual‐function implants. Their physicochemical characteristics can be adapted to control degradation rates and drug release characteristics to fit the clinical requirements of that particular medical field (Ain et al. 2019; Chen et al. 2023; Sergey 2018).

Eggshell‐derived calcium phosphates and processed membranes are promising experimental biomaterial platforms; however, translation requires standardized feedstock and manufacture, chemical and phase characterization, sterilization validation, cytotoxicity and biocompatibility testing, appropriate in vivo studies, device‐specific performance testing, and regulatory review. Clinical benefit should not be claimed until supported by controlled human evidence (Ain et al. 2019; Chen et al. 2023; Sergey 2018; Torres Gouveia et al. 2025).

## 5 Food Applications of Eggshell

### 5.1 Calcium Fortification

Finely processed chicken eggshells can serve as calcium supplements or functional food ingredients because they contain approximately 380 mg calcium per gram. Bartter et al. summarized an estimated fractional calcium absorption of approximately 39%, which is comparable to that of calcium carbonate in the evidence they assessed (Bartter et al. 2018; Lin and Li 2026). Figure 1 shows eggshell‐calcium‐fortified foods.

**FIGURE 1:**
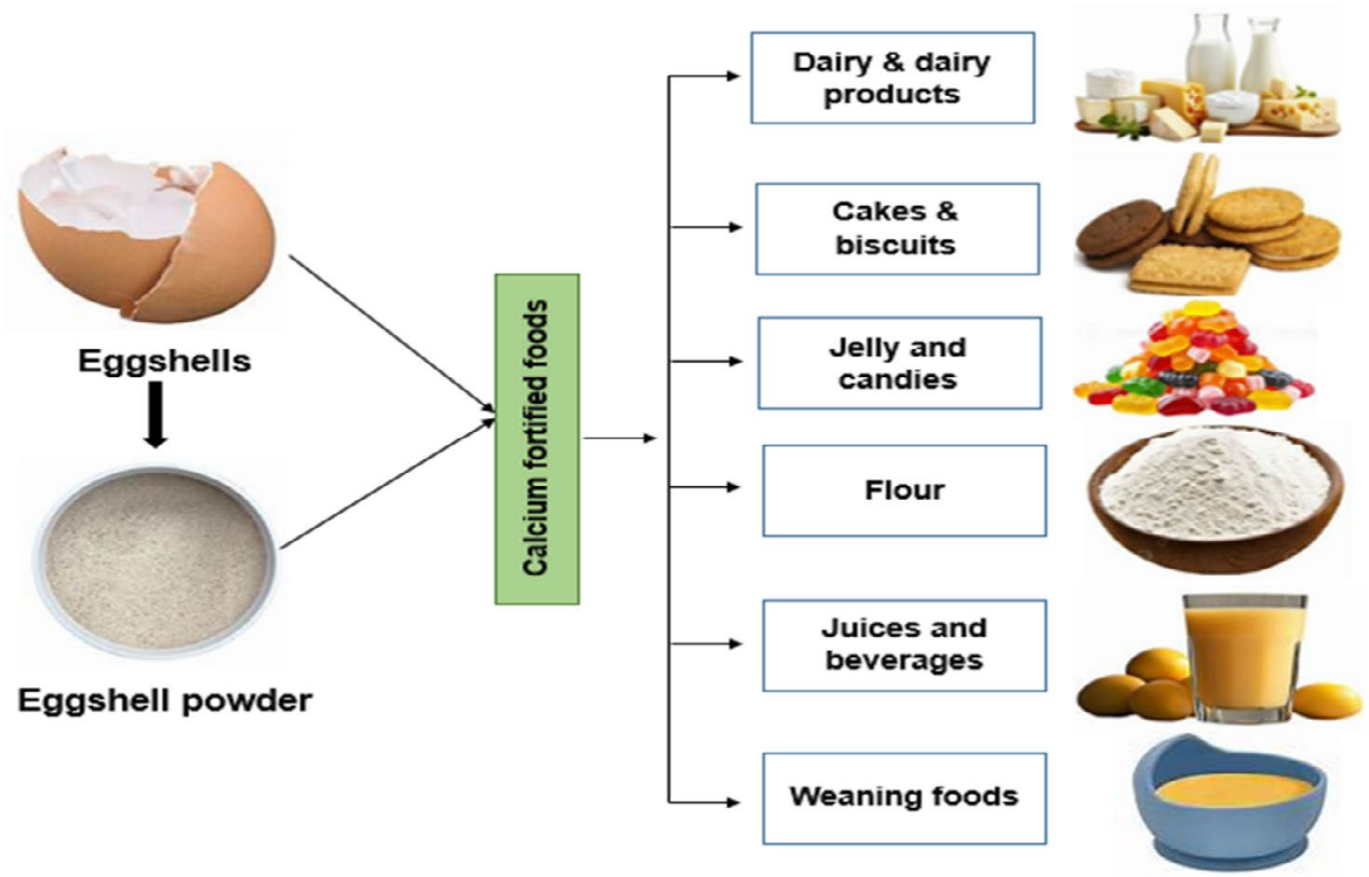
Eggshell calcium fortified foods.

The principal food‐safety concern is contamination associated with residual egg material, including Salmonella. Bartter et al. reported expert agreement that a household sequence involving approximately 10 min of boiling to hard cook the egg followed by about 20 min of cooking the crushed shell in a staple food would address the identified egg‐associated pathogens in that context. This evidence should not be presented as a universal industrial kill step validation. Commercial manufacture requires a validated time, temperature process, prevention of recontamination, microbiological release criteria, traceability, and control of particle size, chemical contaminants, residual protein/allergen risk, and dose (Bartter et al. 2018). In the community, eggshells are initially perceived as waste, but through proper communication, positivity, and making the calcium fortification of eggs safe, the inclusion of eggshell calcium fortification in most communities is mainly accepted, and it provides a fair and locally available intervention (Bartter et al. 2018).

### 5.2 Functional Food Ingredients

Eggshells can be processed using heat treatment to achieve sterility and subsequently crushed to a fine powder that can be used as a functional food ingredient or calcium supplement, such as flours, staple meals, or dietary supplements. Although higher levels of encapsulation present the potential to enhance nutrient stability and release during fortification, studies on the encapsulation of eggshell‐based calcium are limited, which allows for the development of this field further in the future to improve the use of encapsulation in functional foods (Patil et al. 2025).

Eggshell‐derived products to fortify calcium are in line with the broader society ‘s objectiveto reduce the impact of calcium deficiency‐associated illnesses, such as nutritional rickets and osteomalacia. Fortified foods provide an affordable solution to the need to increase the daily calcium intake levels among various populations, particularly in areas with low levels of dairy intake or culturally inappropriate. Nevertheless, there are technical implementation issues, monitoring, and consumer compliance that persist and necessitate joint regulatory and educational efforts (Bartter et al. 2018; Palacios et al. 2020).

### 5.3 Regulation, Quality, and Acceptance

Regulatory status is jurisdiction‐ and product‐specific. For example, the European Commission consultation database classifies calcium salts derived from eggshells within the novel food framework, while animal byproduct rules may also govern collection and processing in the European Union. Future studies should define food‐grade specifications, validate sanitation and shelf stability, quantify calcium bioavailability for the final formulation, assess sensory effects and consumer acceptance, and establish compliant labeling and exposure limits for the intended population (European Commission 2009; Bartter et al. 2018).

Future research should focus on establishing standardized and scalable procedures for cleaning, sterilization, membrane removal, drying, milling, and particle size control of food‐grade eggshell calcium. Validated microbiological safety processes, assessment of residual proteins and potential allergenicity, chemical contaminant testing, shelf‐life evaluation, and batch‐specific quality control criteria are required before commercial application (Bartter et al. 2018). Studies should also determine calcium bioavailability, appropriate intake levels, sensory effects, and stability within the final fortified‐food formulation. Finally, consumer awareness and acceptance studies, together with transparent safety communication, compliant labeling, and jurisdiction‐specific regulatory assessment, will be essential for the successful introduction of eggshell‐derived calcium into functional foods (Arif et al. 2022; Chang et al. 2023).

## 6 Industrial Applications of Eggshell

Materials derived from eggshells have been widely used in various industries, and leveraging their natural calcium carbonate composition and inherent biological properties, Figure 2 shows the industrial applications of eggshell waste.

**FIGURE 2:**
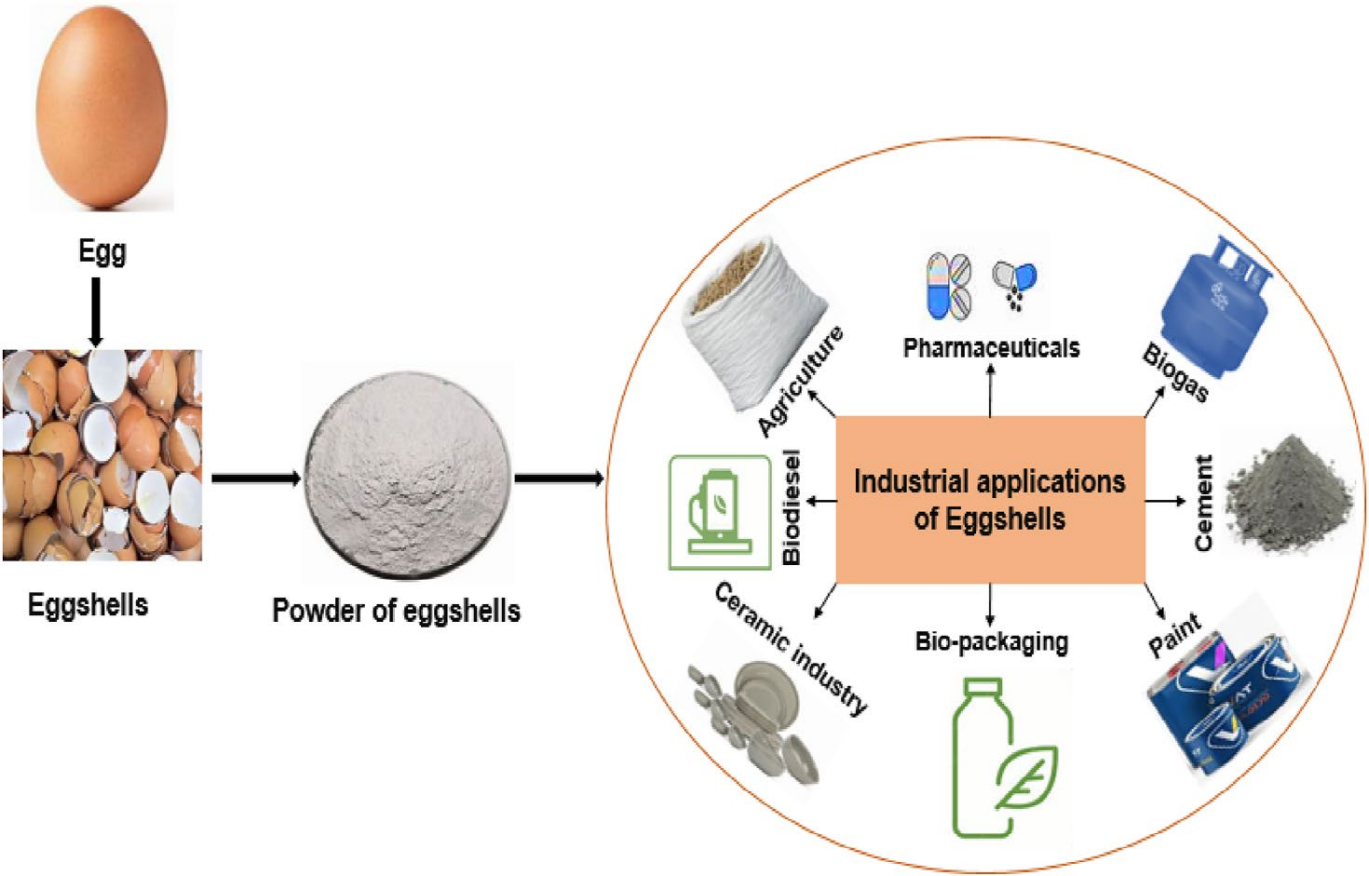
Industrial applications of eggshell waste.

### 6.1 Pharmaceutical Industry

Eggshell‐derived calcium carbonate, HAp, and membrane components have been investigated as starting materials for calcium supplements, drug delivery matrices, biosensors, and regenerative biomaterials. Future pharmaceutical use will require standardized purification, consistent composition, safety evaluation, and application‐specific regulatory validation (Baláž et al. 2021; Torres Gouveia et al. 2025).

### 6.2 Agriculture Industry

In the agriculture sector, ESP is used as an organic calcium‐based soil amendment and substitute for fertilizer. Its mineral composition makes soils more fertile and balanced with the pH, enhancing the growth of plants and providing an environmentally friendly alternative to chemical fertilizers and decreasing the amount of waste in the agricultural system (Hart 2020; Putra et al. 2023).

Eggshell‐derived calcium carbonate and associated nanomaterials are applied in the treatment of wastewater using adsorbents and are effective in the removal of heavy metals and organic pollutants. The nanoscale properties in catalysis and adsorption processes of eggshells are also promoted by mechano‐chemically treated eggshells (Zhang et al. 2019). Moreover, calcined eggshells can be used as catalysts during the production of biodiesel, which contributes to the development of cleaner sources of energy (Baláž et al. 2021; Hart 2020).

### 6.3 Biomaterials and Packaging

In terms of biomaterials and packaging, the use of ESM and powders is done in the creation of biodegradable films and bioplastics to substitute petrochemical‐derived plastics. These films based on egg components have desirable mechanical strength, thermal stability, barrier property, antioxidant, and antimicrobial activities. The active and composite packaging films that incorporate eggshell‐derived materials with other biopolymers and natural additives also enhance performance to facilitate food preservation and food sustainability goals (Abu Ubaidah et al. 2024; Khalid et al. 2024; Shi et al. 2025). Mechanochemical treatment is among the processing methods that increase the nanoscale structure and reactivity of eggshell‐derived materials to allow a broad range of applications in multifunctional catalysts, environmental remediation, biomedical devices, and construction composites. Such methods as thermo‐compression and blending are used to optimize eggshell‐based bio composites for specific applications in industries (Alkaron et al. 2023; Baláž et al. 2021; Khalid et al. 2024).

Overall, eggshell valorization has industrial significance because it can convert an abundant food‐processing byproduct into calcium‐based raw materials and functional additives for multiple manufacturing sectors. However, successful commercialization will depend on reliable waste collection, standardized pretreatment, scalable and energy‐efficient conversion processes, consistent batch quality, economic competitiveness, market demand, and regulatory compliance. Future work should prioritize pilot‐scale validation, life cycle and techno‐economic assessments, and collaboration among egg‐processing facilities, technology developers, manufacturers, and regulatory authorities. Table 3 shows the mechanochemical treatments, processing methods, and product development from eggshells.

**TABLE 3:** Mechanochemical treatments, processing methods, and product development from eggshells.

Grinding input	Testing methods	Outcomes	Developed product	References
Eggshell	Size of particle, SEM, XRD, AFM, mechanical characteristics, and chloride ion permeability	Enhancement of oil well cement's mechanical characteristics and quickening of the hydration process	Cement‐eggshell composite for oil wells	Dawood Salman et al. (2020)
Acetone and eggshells	Raman, SEM, FTIR, and XRD	The impact of different post‐milling sintering temperatures (900°C–1200°C) on the transformation of CaCO_3_‐CaO was examined	Nano‐sizing	Puspitasari et al. (2019)
Eggshell and titanium dioxide	Buffering, SEM, acid tolerant quality, FTIR, TEM, and XRG	Compared to other toothpastes, Colgate, toothpaste and the produced composite cause less damage to the tooth surface	Dentistry and eggshell‐titanium dioxide composite	Onwubu et al. (2019)
Eggshell and titanium dioxide	Buffering, acid tolerant quality, FTIR, TEM, and XRG	The buffering effectiveness was compared to four different toothpastes	Dentistry and eggshell‐titanium dioxide composite	Onwubu et al. (2019)
Eggshell	Distribute the size of particle, SEM, XRD, and EDX	Graphite was present in the eggshell	Micro‐size of the shells	Ononiwu and Akinlabi (2020)
Eggshell and Aluminum oxide	EM, mechanical characteristics, corrosion, and heat expansion	When calcium oxide made from eggshell was added, toughness and ductility decreased but tensile strength, hardness, corrosion resistance, and heat stability increased	Eggshell or aluminum oxide composite	Kumar et al. (2020)
Ethanol and eggshells	Zeta potential, SBET, particle size distribution, SEM, TEM, EDS, FTIR, determining calcium concentration and XRD	During therapy, zeta potential dropped	Nano‐sizing	Huang et al. (2021)
Eggshell membrane and lithium iron silicate	Raman, XPS, TEM, SBET, XRD, and, TA	ESM used as a carbon source to enhance the LFS, ESM composite's electrical characteristics	LFS‐C composite or electrochemistry	Karuppiah et al. (2020)
Eggshell and water	TA, TEM, and XRD	Stearic acid lowers the temperature at which thermal degradation occurs	Nano‐sizing	Villarreal‐Lucio et al. (2019)
Eggshell and the solution of phosphate precursor	SEM, TA, FTIR, and XRD	Wet milling and low‐temperature treatment are used to create pure HA from several precursor using three distinct CaCO_3_ sources	Bio‐ceramics and hydroxyapatite	Cestari et al. (2020)
Eggshells and lithium, nickel, cobalt, manganese, cobalt, oxygen	TA, electrochemical measurements, XRD, FTIR, SEM/EDS, and XPS	Calcium oxide inhibits electrode corrosion and electrolyte dissolution	Lithium, nickel, cobalt, manganese, cobalt, oxygen electrode or electrochemistry covered with calcium oxide	Senthil et al. (2019)
Eggshell and acetone	SEM and XRD	The calcium concentration was enhanced by sintering however the size of the crystallites did not significantly increase	Nano‐sizing	Puspitasari et al. (2019)
Eggshell and rice straw	XRD, SEM, thermodynamics, adsorption kinetics, and the impact of different variables	A balanced eggshell to rice straw ratio demonstrated a maximum sorption capacity of 231 miligram/g	Adsorbent for phosphate ions and wastewater treatment	Liu et al. (2019)
Calcium oxide from eggshell	Fluorescence microscopy, XRD, FTIR, SEM, and bio‐compatibility assessment	Ball milling, mortar and pestle, FDA approved methods, and post‐milling reaction with H_3_PO_4_ are compared	Bio‐ceramics and scaffolds made of β‐tricalcium phosphate	Roopavath et al. (2019)
Eggshell and phosphoric acid	Raman, Tem, XRD, and SEM	Comparing eggshell and cuttlefish bone as a source of calcium without sintering	Bio‐ceramics and hydrxyapatite	Ferro and Guedes (2019)
Eggshell, titanium dioxide and magnesium	TA, SEM, and XRD	Conventional and high energy ball milling comparison	Calcium titanium oxide electronics and ceramics	Cherdchom et al. (2019)
Eggshell and polyvinyl chloride	Titration, FTIR, and XRD	Scalability and comparison of vibratory and planetary milling	Dischlorination and calcium chloride combined with a safe organic matrix	Baláž et al. (2019)
Eggshell and ethanol water	XRD, SEM, TEM, TA, optical microscopy, assessment of mechanical properties and bio‐compatibility	5% weight of nano‐hydroxyapatite, cells proliferate on the fibers	Bioengineering and PLA‐nanohydroxy‐apatite fibers	Apalangya et al. (2019)
Eggshell	Scanning electron microscopy	Using a statistical method, the most crucial element is speed of milling	Bio‐ceramics and hydroxyapatite	Van Hoten et al. (2018)

## 7 Sustainability and Circular Economy Perspective

Waste‐to‐value strategies represent an important change in waste management by transforming discarded materials into useful resources. Related approaches have also been investigated for treating industrial waste streams, including the development of composite flocculants to improve the dewatering of engineering waste slurry (Zhu et al. 2026).

The mineral fraction can be used as a calcium carbonate source, filler, sorbent, catalyst precursor, soil amendment, or raw material for calcium‐based products, whereas the membrane has potential applications in biomedical materials, biocomposites, and other high‐value products. The separate recovery of these fractions can improve resource efficiency and increase the overall value obtained from eggshell waste (Quina et al. 2017; Baláž et al. 2021).

From a circular economy perspective, eggshell valorization can promote industrial symbiosis by connecting poultry farms, hatcheries, restaurants, and food processing facilities with industries requiring calcium‐rich raw materials. Source‐segregated eggshell waste can be locally collected and processed, thereby reducing landfill disposal and partially substituting virgin limestone in suitable calcium‐based applications (Ferraz et al. 2018). A cascaded valorization approach, in which the protein‐rich membrane is recovered before processing the mineral shell, may further increase the economic value obtained from the waste. However, the environmental benefits depend on the selected processing route because washing, sanitation, drying, membrane separation, grinding, and calcination may require water and energy or generate emissions. Therefore, processing methods should be selected according to the intended application while minimizing resource consumption and preserving the value of both eggshell fractions.

## 8 Commercialization and Industrial Translation: Challenges and Future Directions

Commercial translation of eggshell waste valorization requires a reliable supply chain and standardized preprocessing procedures. Industrial eggshell waste can vary in membrane content, moisture, residual organic matter, microbial load, and mineral composition depending on the egg source and collection conditions. Therefore, collection, washing, membrane separation, sanitation, drying, milling, and storage conditions should be standardized to obtain reproducible raw materials. Product specifications should include moisture content, particle‐size distribution, CaCO_3_ or CaO content, residual protein, microbial limits, and potentially harmful contaminants. Batch‐to‐batch quality control is particularly important because different applications require different levels of purity and processing severity (Torres Gouveia et al. 2025; Vandeginste 2021). A major limitation is the absence of standardized processing and quality control procedures. Eggshell characteristics may vary with poultry species, diet, age, and production conditions, while differences in collection, washing, membrane separation, sterilization, drying, grinding, particle‐size control, calcination, and chemical conversion can affect the composition and performance of the final product. Standardized specifications are therefore required for microbial quality, residual organic matter, mineral composition, particle size, phase purity, and possible contaminants. Processing requirements must also be matched to the intended application because food‐grade calcium powder, biomedical HAp, environmental adsorbents, and industrial fillers require different safety and performance criteria (Therdthai et al. 2023; Vandeginste 2021).

Clinical validation is primarily a challenge for biomedical applications of eggshell‐derived materials, including eggshell‐membrane scaffolds, HAp implants, wound dressings, and drug‐delivery systems. Although many formulations have demonstrated promising physicochemical properties and in vitro biological performance, evidence from standardized animal studies and controlled human clinical studies remains limited. Clinical translation will require reproducible manufacturing, sterilization validation, biocompatibility and toxicity assessment, long‐term performance evaluation, and compliance with the regulatory requirements applicable to medical devices or therapeutic products (Mensah et al. 2023; Torres Gouveia et al. 2025).

Scaling up eggshell valorization requires reliable collection systems and sufficient processing capacity to handle large and variable volumes of material. Commercial feasibility may be affected by transportation, storage, cleaning, membrane separation, drying, milling, sterilization, and product purification costs. High‐temperature processes, particularly calcination, also require substantial energy and may release CO_2_ during the conversion of CaCO_3_ to CaO. Therefore, scale‐up must maintain consistent product composition, particle size, phase purity, and functional performance across production batches. Pilot‐scale studies, process optimization, TEA, and life‐cycle assessment are needed to determine whether the environmental and economic benefits are maintained at the industrial scale (Ngayakamo and Onwualu 2022; Vandeginste 2021).

Consumer acceptance is especially important for eggshell‐derived food ingredients, calcium supplements, cosmetics, and biomedical products. Acceptance may be affected by concerns about microbial contamination, product purity, allergenicity, safety, and the perception of consuming or using a waste‐derived material. Studies of eggshell‐fortified foods indicate that consumer acceptance is influenced by awareness, taste, texture, and the concentration of ESP incorporated into the product. Transparent communication regarding the source of the material, decontamination procedures, quality‐control testing, nutritional or therapeutic benefits, and regulatory approval may therefore improve consumer confidence (Arif et al. 2022; Chang et al. 2023).

Successful commercialization will require application‐specific processing standards, reliable quality control systems, regulatory guidance, safety and performance validation, and economically feasible scale‐up. Future studies should prioritize pilot‐scale manufacturing, batch‐to‐batch reproducibility, life‐cycle and techno‐economic assessments, and collaboration among egg‐processing industries, researchers, manufacturers, regulators, and end users. These measures will help integrate eggshell valorization into safe and commercially viable circular‐economy systems.

## 9 Conclusion

Eggshell waste represents a sustainable biomaterial resource with potential value across food, biomedical, environmental, and industrial sectors. Its high calcium carbonate content, trace mineral composition, and organic matrix enable its conversion into functional materials, while ESM components, including collagen and GAGs, provide additional opportunities for specialized biomedical applications. Overall, eggshell valorization can reduce poultry processing waste, replace some conventional mineral resources, and support the development of value‐added products within a circular bioeconomy.

Nevertheless, the transition from laboratory‐scale research to practical application remains constrained by variability in eggshell composition arising from differences in poultry species, diet, age, and processing history. The absence of standardized procedures for collection, cleaning, membrane separation, sterilization, particle‐size control, and chemical conversion can also affect the quality and reproducibility of eggshell‐derived products. Furthermore, possible microbial contamination, residual organic matter, trace‐element variability, and process‐derived impurities require comprehensive safety assessment. Biomedical, food, and environmental applications must therefore be supported by appropriate toxicological, biological, and performance validation, together with compliance with application‐specific regulatory requirements.

Future research should prioritize scalable, energy‐efficient, and environmentally responsible processing technologies that provide consistent product quality. Pilot‐scale production, comparative life cycle and techno‐economic assessments, long‐term safety studies, clinical or field validation, and standardized quality‐control criteria are required before widespread commercialization. The integration of eggshell collection and valorization systems with poultry farms, food processing facilities, material manufacturers, and waste management networks will be essential for establishing reliable circular economy supply chains. With appropriate processing standardization, validation, and regulatory oversight, eggshell waste can become a valuable secondary resource that contributes to waste reduction, resource conservation, and sustainable economic development.

## Author Contributions

**Farhang Hameed Awlqadr:** writing – review and editing, writing – original draft, visualization, validation, methodology. **Iffat Ullah:** writing – review and editing, writing – original draft, visualization, validation, investigation. **Muhammad Tayyab Arshad:** writing – original draft, writing – review and editing, visualization, validation, conceptualization. **Mamoona Shehzadi:** writing – original draft, writing – review and editing, project administration, formal analysis, validation. **Ali Ikram:** writing – original draft, writing – review and editing, project administration, formal analysis, data curation. **Meerab Sajid:** writing – original draft, investigation, validation, formal analysis, writing – review and editing. **Mohammed Ali Al‐Duais:** writing – original draft, writing – review and editing, project administration, data curation, resources. **Md. Sakhawot Hossain:** writing – original draft, writing – review and editing, resources, supervision, data curation.

## Funding

This study received no external funding.

## Ethics Statement

This study did not involve human or animal subjects.

## Consent

This study did not involve human subjects.

## Conflicts of Interest

The authors declare no conflicts of interest.

## Data Availability

The data that support the findings of this study are available from the corresponding author upon reasonable request.
